# Viability and proliferation of endothelial cells upon exposure to GaN nanoparticles

**DOI:** 10.3762/bjnano.7.124

**Published:** 2016-09-23

**Authors:** Tudor Braniste, Ion Tiginyanu, Tibor Horvath, Simion Raevschi, Serghei Cebotari, Marco Lux, Axel Haverich, Andres Hilfiker

**Affiliations:** 1 National Center for Materials Study and Testing, Technical University of Moldova, bv. Stefan cel Mare 168, MD-2004 Chisinau, Republic of Moldova; 2 Leibniz Research Laboratories for Biotechnology and Artificial Organs (LEBAO), Department of Cardiothoracic, Transplantation and Vascular Surgery, Hannover Medical School, Carl Neuberg Str. 1, D-30625 Hannover, Germany; 3 Department of Physics and Engineering, State University of Moldova, str. Alexe Mateevici 60, MD-2009 Chisinau, Republic of Moldova

**Keywords:** endothelial cells, GaN nanoparticles, proliferation, surface functionalization

## Abstract

Nanotechnology is a rapidly growing and promising field of interest in medicine; however, nanoparticle–cell interactions are not yet fully understood. The goal of this work was to examine the interaction between endothelial cells and gallium nitride (GaN) semiconductor nanoparticles. Cellular viability, adhesion, proliferation, and uptake of nanoparticles by endothelial cells were investigated. The effect of free GaN nanoparticles versus the effect of growing endothelial cells on GaN functionalized surfaces was examined. To functionalize surfaces with GaN, GaN nanoparticles were synthesized on a sacrificial layer of zinc oxide (ZnO) nanoparticles using hydride vapor phase epitaxy. The uptake of GaN nanoparticles by porcine endothelial cells was strongly dependent upon whether they were fixed to the substrate surface or free floating in the medium. The endothelial cells grown on surfaces functionalized with GaN nanoparticles demonstrated excellent adhesion and proliferation, suggesting good biocompatibility of the nanostructured GaN.

## Introduction

The development of new multifunctional and hybrid “smart materials” for biological and medical applications is of paramount importance [1]. Biomaterial research is closely associated with the development of chemical/biochemical sensors, hydrogels, membranes, and artificial organs, and is utilized in applications such as the recognition of premature tissue damage and dispensing of medications. Nature supplies many examples of biomimetic materials in the form of organic/inorganic components such as bone, teeth, and muscle. Based on biological examples, new and innovative biological materials can be designed through self-organization or direct structuring. Focusing on the methods involving biointerfaces and the connection between technology and cells, further developments are being made in bioactive material targets, regeneration of natural tissue [2], and the acceleration or delay of biological or biochemical processes [3]. Nanoparticle-based systems are the best choice for biomedical applications not only due to their small size (from 1 to 100 nm), but also due to their potency, which results from their high surface area for active molecules.

Over the last decade, many studies have been performed on the interaction between living cells and different types of nanoparticles of specific shape, surface charge, and chemical composition [4]. For example, elongated, rod-like gold (Au) nanoparticles affect the viability of endothelial cells more than spherical ones, which have better biocompatibility [5]. However, multiwalled carbon nanotubes (MWCNTs) with a diameter less than 30 nm are less toxic than particles larger than 50 nm [6].

Nearly all current and future biomedical applications implicate intravascular transport of nanoparticles, thereby blood is the predominant carrier for nanoparticles and nanoparticle clusters. The first barrier to penetrate the tissue after intravascular application of nanoparticles are endothelial cells. A single monolayer of these cells covers the inner surface of blood vessels, thus playing the role of an active interface between the circulating blood and the tissue [7–8]. As soon as nanoparticles are in the circulatory system, they likely first interact with endothelial cells. By acting on endothelial cell function, they may not only affect existing blood vessels, but also the ability to form new blood vessels (a process called angiogenesis). Being crucial for the growth and development of a living organism, angiogenesis has an important role in the wound healing process, where new blood vessels are being formed from pre-existing ones [9]. It is widely believed that endothelial cells communicate to each other and, as a result of their communication and redistribution, form hollow capillary tubes and thus develop new blood vessels via ramification of the existing ones. The development of the blood vessel wall after the capillary formation discloses the efficient intercellular communication [10]. Tissue engineering and artificial organ development are also emerging fields that involve vasculature building to supply the cells with nutrients. The manipulation of angiogenesis might also result in inhibition of blood vessel development. The ability to stimulate or inhibit the formation of blood vessels is of paramount importance for the development of new organs and it is a challenge for controlled tumor retention. The understanding of endothelial cell biology is a prerequisite to fight cancer growth because all tumors are dependent on the vasculature. A detailed study of the interaction of endothelial cells with nanoparticles is necessary for future applications to manipulate endothelial cell function. Many studies have tried treating endothelial cells with peptide-coated nanoparticles, with the final outcome dependent upon the surfactant bound to the nanoparticle rather than the nanoparticle itself [11–12]. We propose the use of “smart materials” for such purposes. GaN could be a good candidate material due to its chemical inertness and, in particular, its piezoelectric properties, which opens the possibility of it being able to transmit an electrical signal to cells. This might be used to activate cells receptors through simple external activation (e.g., via an ultrasound field). In comparison to other materials intensively investigated in this area (such as boron nitride nanotubes [13], barium titanate [14], or hydroxyapatite [15]), nanoscale GaN, in addition to its biocompatibility, shows strong enhancement of the piezoelectric properties compared to the bulk material [16–17]. At the same time, the huge potential of integration of GaN in nano-optoelectonic devices opens up the possibility to test the material in lab-on-a-chip technologies.

In this study, porcine aortic endothelial cells were investigated in direct contact with GaN nanoparticles. GaN is a compound semiconductor material, widely used in optoelectronics, in particular in industrial production of UV solid-state lasers and light-emitting diodes. This material has many remarkable characteristics, including piezoelectric properties, high thermal stability, radiation hardness, and excellent chemical inertness, which make it promising for biomedical applications. Recently, it was shown that GaN does not require surface treatment for biocompatibility [18]. Bulk GaN is nontoxic for cells with or without peptide functionalization of the surface, with only very low amounts of gallium being released during the cell cultivation process [19]. There is, however, limited knowledge about the biocompatibility of nanostructured GaN and the impact of GaN nanoparticles on living cells.

## Results

**Figure 1:**
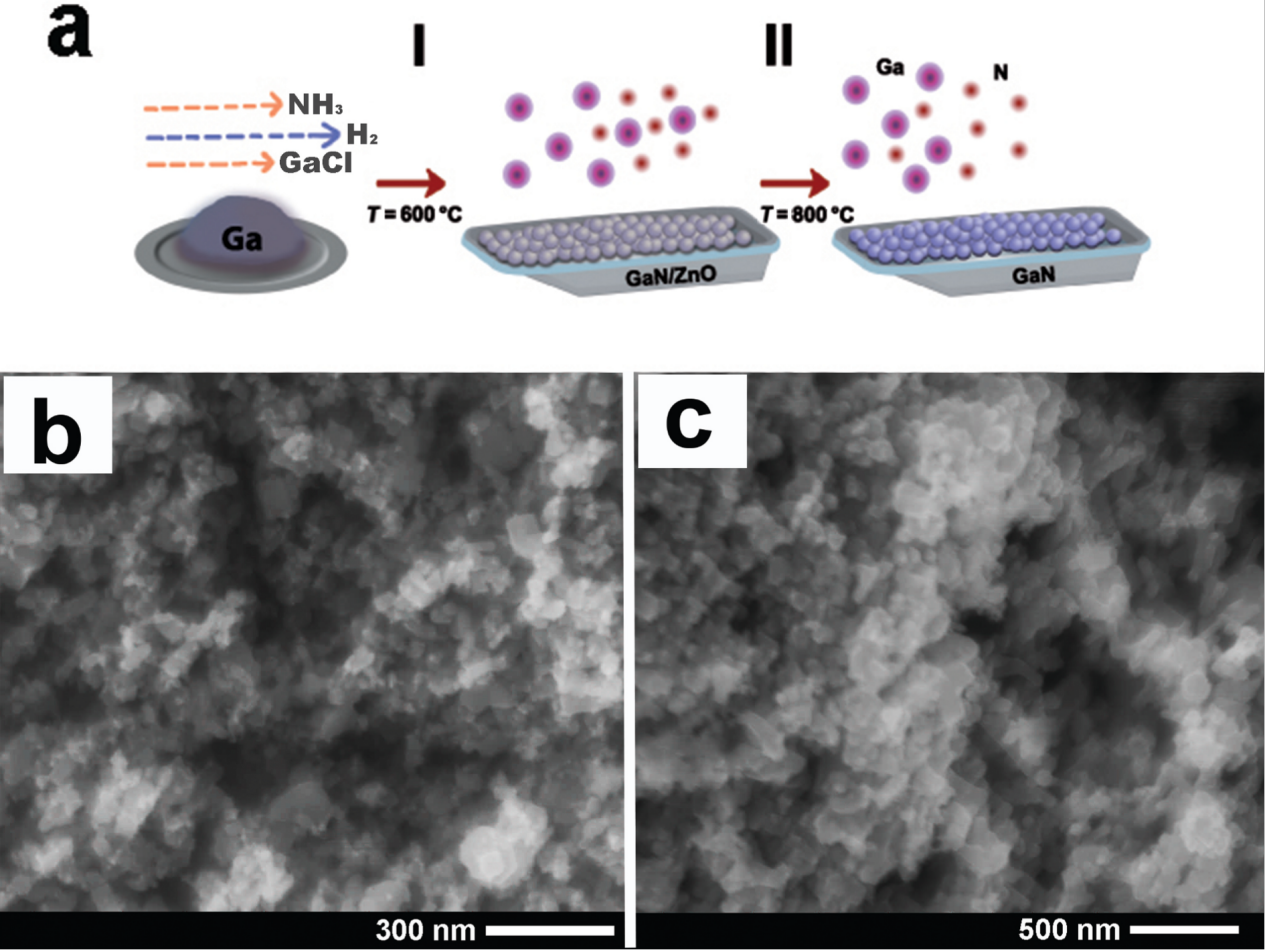
Schematic representation of the GaN growth process on ZnO nanoparticles. (a) I – the nucleation process of GaN at low temperature, II – the high temperature step for layer growth and sacrificial ZnO core decomposition. SEM images of (b) ZnO nanoparticles and (c) GaN nanoparticles grown on a sacrificial layer of ZnO.

GaN nanoparticles with diameters ranging from 50 to 100 nm were synthesized on a sacrificial layer of ZnO nanoparticles, as depicted in Figure 1. According to energy dispersive X-ray (EDX) measurements, approximately 2% of ZnO remained on the GaN nanoparticles after thermal treatment in hydrogen flow (Supporting Information File 1, Figure S1). The further removal of ZnO was not possible despite higher temperatures and longer incubation time under hydrogen flow. According to the chemical analysis of the culture medium after incubation with GaN nanoparticles, a very low concentration of Zn was detected, suggesting the high chemical stability of the compound (Supporting Information File 1, Figures S2 and S3). Therefore, the release of toxic Zn from the nanostructures is unlikely.

**Figure 2:**
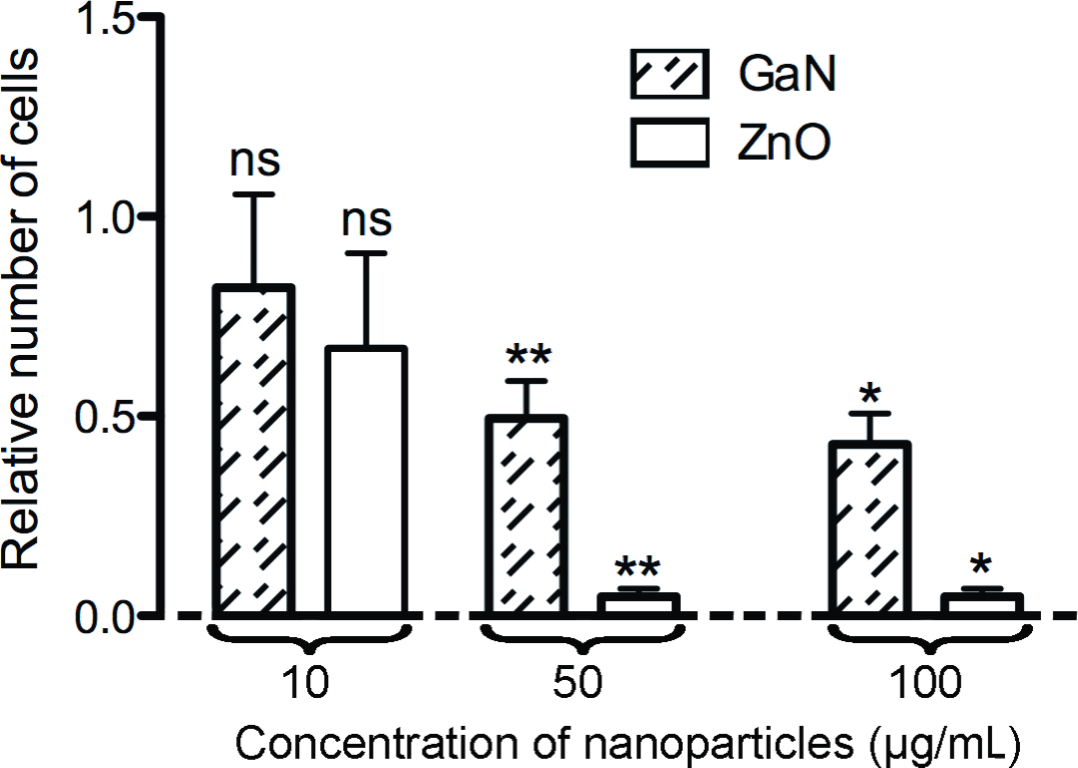
Relative number of endothelial cells after 3 days of incubation with 10 µg/mL, 50 µg/mL, or 100 µg/mL GaN or ZnO nanoparticles compared to untreated cells. Values are expressed as mean ± standard deviation of 3 independent experiments with 3 replicates. ns: nonsignificant, * *p* < 0.05, ** *p* < 0.01.

Firstly, the effect of an increasing concentration of free GaN nanoparticles on porcine aortic endothelial cell growth was determined (Figure 2). As a positive control for toxicity, the cells were also exposed to corresponding concentrations of free ZnO nanoparticles. After a three-day incubation period, there was a significant decrease in the number of adherent endothelial cells after exposure to 50 and 100 µg/mL ZnO nanoparticles. In contrast, there was no significant decrease in the number of adherent endothelial cells after a three-day exposure to a concentration of 10, 50, or 100 µg/mL GaN nanoparticles.

**Figure 3:**
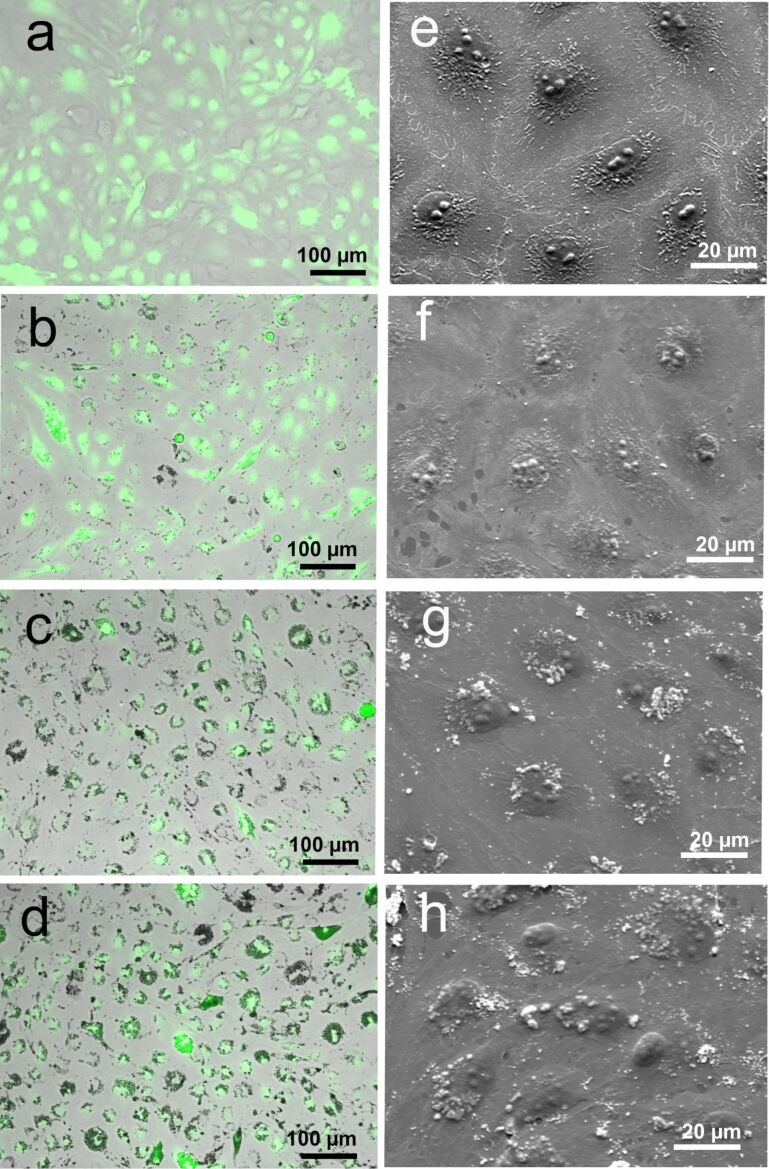
GFP labeled porcine aorta endothelial cells after three days of incubation with free GaN nanoparticles. Fluorescence (a–d) and SEM (e–h) images show the distribution of GaN nanoparticles and the morphological changes to endothelial cells upon exposure to GaN nanoparticles. The cells were exposed to either 10 µg/mL (b,f), 50 µg/mL (c,g), or 100 µg/mL (d,h) of GaN nanoparticles. Images a and e are negative controls treated with media alone.

The uptake of free GaN nanoparticles was also microscopically visualized (Figure 3) and found to be correlated with increasing concentration of GaN nanoparticles. At higher concentrations, the cell morphology was preserved (Figure 3e–h) as assessed by SEM.

**Figure 4:**
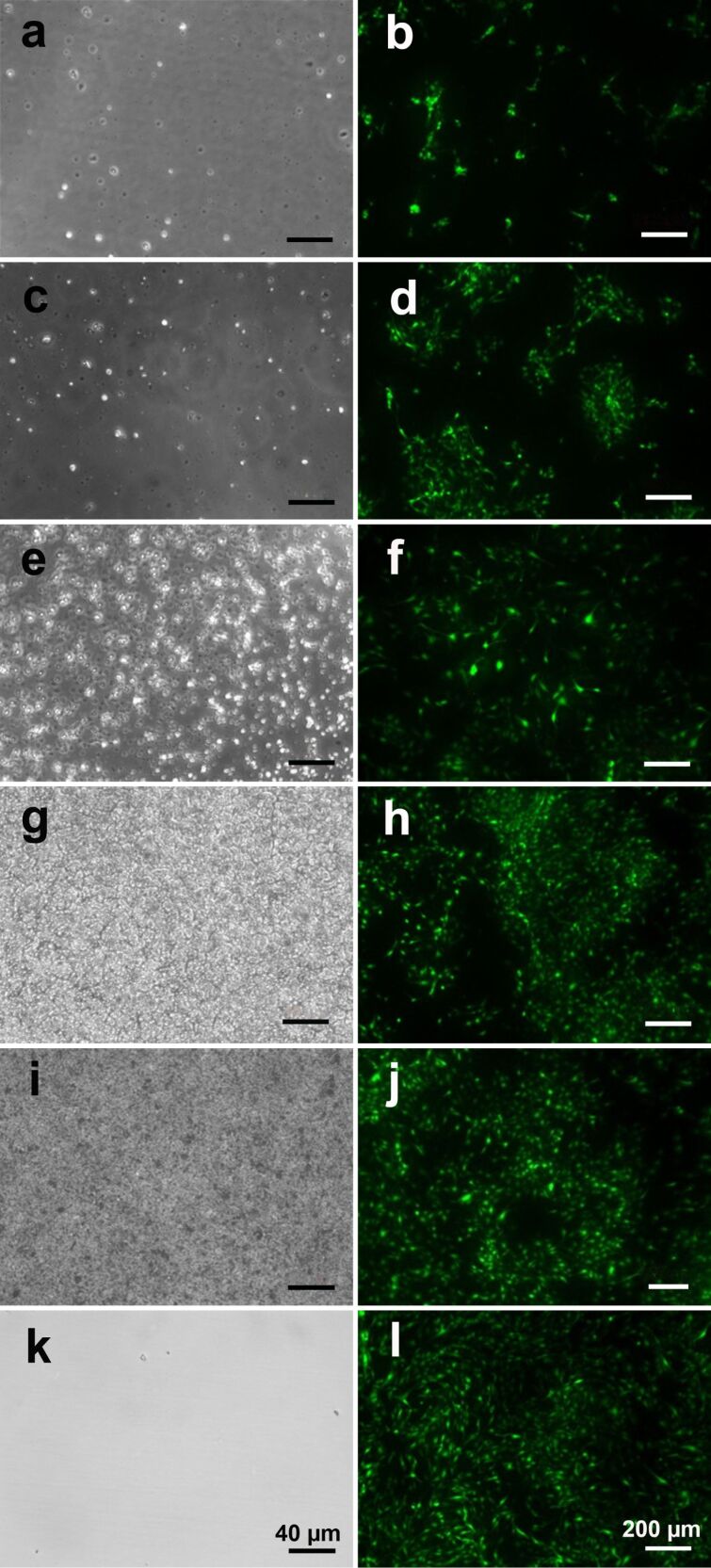
Porcine aortic endothelial cells cultured on GaN-functionalized glass coverslips. (a,c,e,g,i) Coverslips coated with silicone and treated with 0, 0.5, 5, 50, or 250 µg/cm^2^ GaN nanoparticles, respectively. (b,d,f,h,j) The GFP labeled porcine endothelial cells cultured on the 0, 0.5, 5, 50, or 250 µg/cm^2^ GaN-functionalized surfaces. (k,l) Cells seeded on uncoated, untreated glass coverslips.

**Figure 5:**
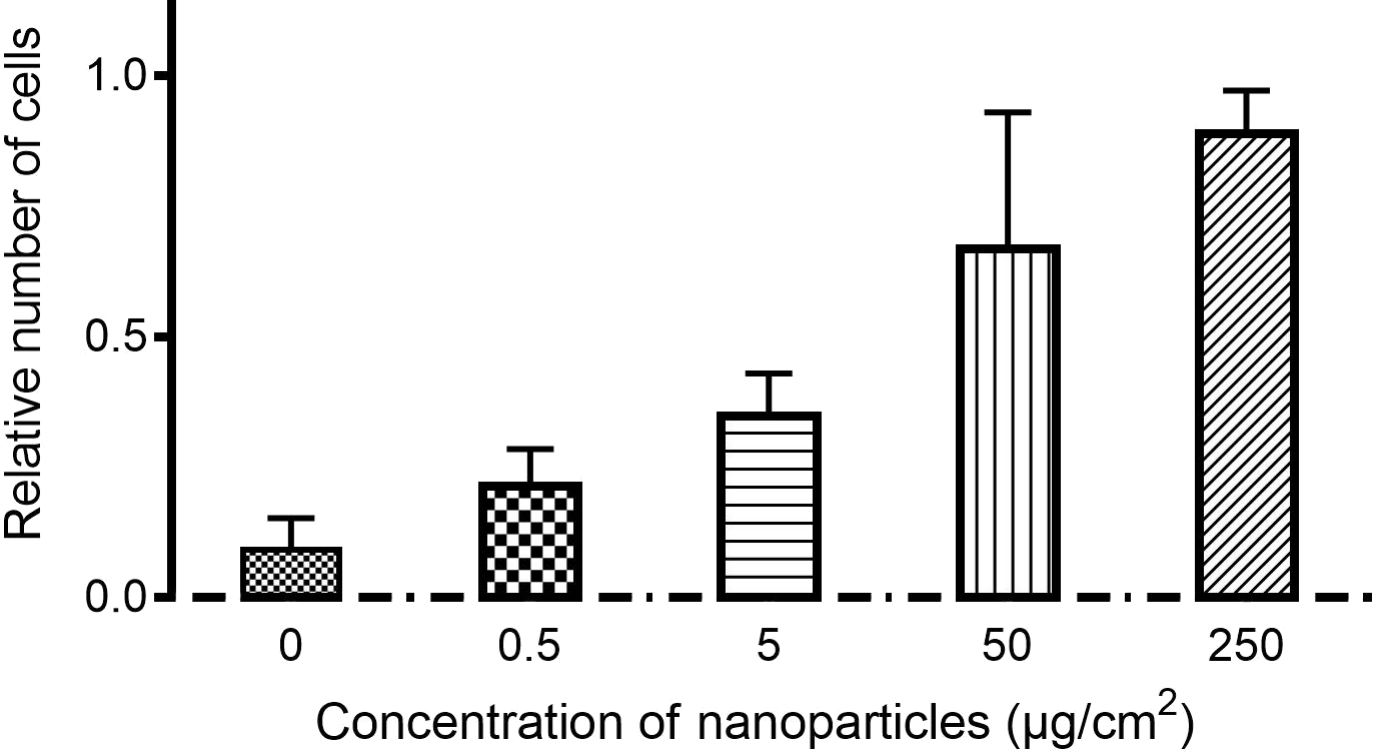
Relative number of adherent endothelial cells after 3 days of incubation with silicone-coated glass coverslips functionalized with GaN nanoparticles compared to uncoated, untreated coverslips. Values are expressed as mean ± standard deviation of 3 independent experiments with 2 replicates.

Secondly, the adherence and growth of porcine aortic endothelial cells was examined on GaN-functionalized glass coverslips. The glass coverslips were coated with silicone alone (negative control for cellular adherence, Figure 4a,b) or silicone plus GaN nanoparticles (Figure 4c–h). Figure 4b shows minimal adherence of endothelial cells to nonfunctionalized silicone. GaN nanoparticles of increasing concentration were coated on silicone and microscopically visualized (Figure 4c,e,g,i). Endothelial cell adherence was assessed on coverslips functionalized with different concentrations of GaN nanoparticles (Figure 4 and Figure 5). The relative number of cells after three days of incubation on surfaces functionalized by GaN nanoparticles was quantified and compared to the number of cells cultivated on clean glasses (Figure 5). The increase in the density of GaN nanoparticles stimulates the processes of cellular adhesion and growth.

## Discussion

Upon incubation of endothelial cells with and without free GaN nanoparticles we observed that untreated cells displayed higher mobility and a higher rate of division in comparison to cells in contact with nanoparticles. We hypothesize that cells could be affected by the mechanical load of incorporated nanoparticles, which could result in reduced migration and slowed proliferation process with increasing quantities of incorporated nanoparticles [20]. When nanoparticles were added after the cellular layer reached 50% confluence, we clearly observed how the same concentration of ZnO nanoparticles killed cells in a relatively short period of time, while similarly sized GaN nanoparticles, applied at the same concentration, were taken up by endothelial cells, which continued their proliferation process. It is widely believed that the toxicity of ZnO nanoparticles is a result of chemical instability and involves an increase of Zn^2+^ in the culture medium [21].

After three days of incubation, GaN nanoparticles were observed in close association with porcine endothelial cells. These findings are promising with regard to targeting endothelial cells with nanoparticles [22]. Cell targeting with nanoparticles could be applied in diseases affecting vascular structures. In such a case, nanoparticles could be injected in a highly vascularized area, like in some eye diseases that affect the posterior eye, where intraocular injection procedures could harm healthy tissue and cause vision-threatening complications [23–25]. Cellular therapies that involve targeting of nanoparticle-loaded endothelial cells to sites of arterial injury using an external electric or magnetic field, could speed up re-endothelialization by promoting integration into the new endothelial layer. Since each cell division reduces the number of the incorporated GaN nanoparticles by 50%, the load of GaN nanoparticles in a single cell diminishes with time.

The growth of endothelial cells on top of fixed nanoparticles avoids the internalization process of nanoparticles by cells. Surface functionalization is important to prevent biofilm formation on implants or prostheses and can improve the endothelialization process for other applications. It was recently shown that GaN nanoparticles could inhibit biofilm formation, being most effective against Gram-negative bacterial species [26]. Endothelial cells cultivated on surfaces functionalized by GaN nanoparticles show a different behavior compared to cells incubated with floating GaN nanoparticles. Endothelial cells easily attach to fixed nanoparticles without being influenced by the high concentration of nanoparticles. Rema^®^Sil silicone is a biocompatible material to which cells cannot efficiently attach. We observed minimal adherence of endothelial cells to clean silicone surfaces after three days of incubation, while an increasing number of attached cells were observed on surfaces functionalized by nanoparticles. The number of attached cells increases when the density of nanoparticles fixed on top of silicone surfaces increased and no signs of toxicity from the substrate were observed (Figure 5). A different outcome was observed for cells incubated with free-floating nanoparticles, where the total number of cells after three days of incubation tended to decrease at high nanoparticle concentrations. The mechanism underlying the reduction of cell number upon incubation with increasing concentration of free GaN nanoparticles in the culture medium is not yet completely understood. It is widely believed that the large surface area of nanoparticles influences cells to generate reactive oxygen species that play a role in cell killing under high nanoparticle concentrations even though the material is chemically stable [27–28].

The topography of the surface on which endothelial cells are cultivated seems to be less important than the surface chemistry. In our previous tests we noticed that endothelial cells proliferate well on the surface of both bulk material and photo-electrochemically nanostructured layers of GaN. Binding GaN nanoparticles on the silicone surface allows adhesion of the cells to the functionalized surface, which proves to be more susceptible for them. In contrast to this situation, the cells take up all the GaN nanoparticles floating in the medium, thus burning energy via the endocytosis process and slowing down the proliferation activity.

## Conclusion

We found that the interaction of porcine endothelial cells with GaN nanoparticles strongly depends on the state of the nanoparticles: that is, whether they are fixed to the substrate surface or whether they float freely in the medium. The endothelial cells attract free nanoparticles and, depending on the nanoparticle concentration, the cellular activity is slowed down, resulting in lower cellular mobility. Nevertheless, cellular proliferation does not seem to be affected as cells continue to divide even when a relatively large number of nanoparticles are internalized. Fixing nanoparticles to nonadherent, biocompatible silicone, we showed an enhancement in cell adhesion on surfaces functionalized by nanoparticles and signs of toxicity were not observed even with an increased density of fixed nanoparticles, thus indicating to a high biocompatibility of the GaN nanomaterial. By further exploration of the semiconducting and piezoelectric properties of GaN nanoparticles, the characteristics of functionalized surfaces of implants or prosthesis could be easily modulated from the exterior (e.g., with an ultrasound field), making surfaces either more or less attractive for desired types.

## Experimental

### Preparation of GaN nanoparticles

Thin layers of GaN were grown on ZnO nanoparticles by hydride vapor phase epitaxy (HVPE) in two stages, as previously described [29]. Metallic gallium, ammonia (NH_3_) gas, hydrogen chloride (HCl) gas, and hydrogen (H_2_) were used as source materials and carrier gases. In the source zone, GaCl was formed as a result of chemical reactions between gaseous HCl and liquid Ga. GaCl and NH_3_ gas reacted with each other in the react zone, where the initial temperature was kept at 600 °C for 10 min to initiate nucleation of GaN on the surface of ZnO. The temperature was then increased to 800 °C for another 10 min to grow a GaN layer on the ZnO nanoparticles. At 800 °C, along with the growth of GaN, the ZnO core decomposed due to hydrogen flow in the reaction chamber. In the process of GaN growth, the HCl, NH_3_, and H_2_ flow rates were 15 sccm, 500 sccm, and 3600 sccm, respectively. The resulting nanomaterial was used in culture with porcine aortic endothelial cells. Figure 1a shows the schematic of the fabrication process of GaN nanoparticles and SEM pictures of initial ZnO and GaN composite nanomaterial are presented in Figure 1b and Figure 1c.

### Functionalization of surfaces with GaN nanoparticles

Cellular nonadherent, biocompatible silicone (Rema^®^Sil) was used for the nanoparticle fixation process. Rema^®^Sil components A and B were mixed 1:1 and the mixture was spread evenly on glass coverslips by spin coating at 300*g*. Immediately after coating, nanoparticles suspended in deionized water were added on top of the silicone-covered coverslips. The coated coverslips were dried for 24 h at 60 °C and sterilized at 180 °C for 4 h. Before cell seeding, the coverslips were rinsed with sterile, deionized water to remove unattached nanoparticles.

### Isolation and culture of porcine aortic endothelial cells

Porcine aortic endothelial cells were isolated from aortas harvested at the local slaughterhouse as previously described [30]. The cells were cultivated in Endothelial Growth Factor Medium™ 2 (EGM™-2, Lonza) and incubated in a standard incubator at 37 °C with 5% CO_2_. The cells were split with TripLE^TM^Select(1×) (Gibco^®^). All experiments were performed with cells between passage 5 and 9. The cells were labeled with green fluorescence protein (GFP) by lentiviral transduction as described elsewhere [31].

### Interaction of porcine aortic endothelial cells with nanoparticles

Two different approaches of nanoparticle interaction with living cells were tested. In the first approach, GaN nanoparticles were suspended in EGM™-2 medium and incubated with endothelial cells (25,000 cells/well in 1 mL of medium) for three days in 24 well plates. Three different concentrations of GaN nanoparticles were assayed: 10 µg/mL, 50 µg/mL, and 100 µg/mL. The cells incubated with corresponding concentrations of ZnO nanoparticles served as a positive control for toxicity, whereas medium alone served as a negative control. The cells were seeded either in the presence of nanoparticles in the medium, or a nanoparticle-supplemented medium was added to the endothelial cells after reaching 50% confluence. The second approach involved cultivation of endothelial cells (25,000 cells/well) on coverslips functionalized by GaN nanoparticles. In contrast to the first approach, the concentrations of nanoparticles tested were 0.5 µg/cm^2^, 5 µg/cm^2^, 50 µg/cm^2^, and 250 µg/cm^2^.

### Cell counting

The cells were fixed in 4% paraformaldehyde for 10 min, washed with PBS, and stained with DAPI (1:7500 diluted in PBS) for 10 min. Three random fields of view were photographed from each well with a high-resolution camera installed on a fluorescence microscope (Zeiss). Computer-assisted software, DotCount v1.2 [32], was used for quantifying the relative number of cells in every well and compared to the control.

### Cell preparation for scanning electron microscopy

The morphology of aortic endothelial cells cultivated in the presence of GaN nanoparticles was studied using a Philips-505 scanning electron microscope (SEM). Before imaging, the cells were fixed, dehydrated, dried, and a thin metallic layer was sputtered on top of them in order to avoid charging effects during scanning. The fixation process was performed at 4 °C in 2.5% glutaraldehyde for 12 h after the samples were kept in 0.2 M sodium cacodylate buffer for 24 h. The dehydration process involved incubation in gradually increasing acetone concentrations from 30% to 100% at room temperature. Thereafter, the acetone was gradually replaced by liquid CO_2_ at 10 °C in a critical drying point machine (BAL-TEC 030 CPD). In order to bring the sample from the liquid to the gas phase without crossing a phase boundary, it was driven through the “supercritical region”. The pressure and temperature were raised above the critical point where the distinction between gas and liquid ceases to exist. Lowering the pressure, with the temperature still above the critical level, the liquid is transformed into gas without crossing the interface. The final step before imaging involved sputtering the surface of samples with 10 nm of elementary gold (Au) using a Polaron SEM coating system.

### Statistics

All data are given as mean ± standard deviation. The statistical analysis was performed with the Prism software. Differences between groups were evaluated by Student’s *t*-test or ANOVA followed by Bonferoni as appropriate. A value of *p* ≤ 0.05 was considered to be significant.

## Supporting Information

File 1 Details regarding nanoparticle characterization, chemical analysis measurements and schematics of the surface functionalization process with nanoparticles.
