# Supplementary material for: Viability and proliferation of endothelial cells upon exposure to GaN nanoparticles
Source: Beilstein J Nanotechnol. 2016 Sep 23;7:1330–7. doi: 10.3762/bjnano.7.124 (PMC5082458; doi:10.3762/bjnano.7.124)
Supplement: File 1 — Details regarding nanoparticle characterization, chemical analysis measurements and schematics of the surface functionalization process with nanoparticles. [file Beilstein_J_Nanotechnol-07-1330-s001.pdf]

Supporting Information

for

Viability and proliferation of endothelial cells upon exposure to GaN nanoparticles

Tudor Braniste[1,2], Ion Tiginyanu[1], Tibor Horvath[2], Simion Raevschi[3], Serghei Cebotari[2], Marco Lux[2], Axel Haverich[2] and Andres Hilfiker[2,*]

Address: [1]National Center for Materials Study and Testing, Technical University of Moldova, bv. Stefan cel Mare 168, MD-2004 Chisinau, Republic of Moldova, [2]Leibniz Research Laboratories for Biotechnology and Artificial Organs (LEBAO), Department of Cardiothoracic, Transplantation and Vascular Surgery, Hannover Medical School, Carl Neuberg Str. 1, D-30625 Hannover, Germany and [3]Department of Physics and Engineering, State University of Moldova, str. Alexe Mateevici 60, MD-2009 Chisinau, Republic of Moldova

Email: Andres Hilfiker* - hilfiker.andres@mh-hannover.de

* Corresponding author

Details regarding nanoparticle characterization, chemical analysis measurements and schematics of the surface functionalization process with nanoparticles

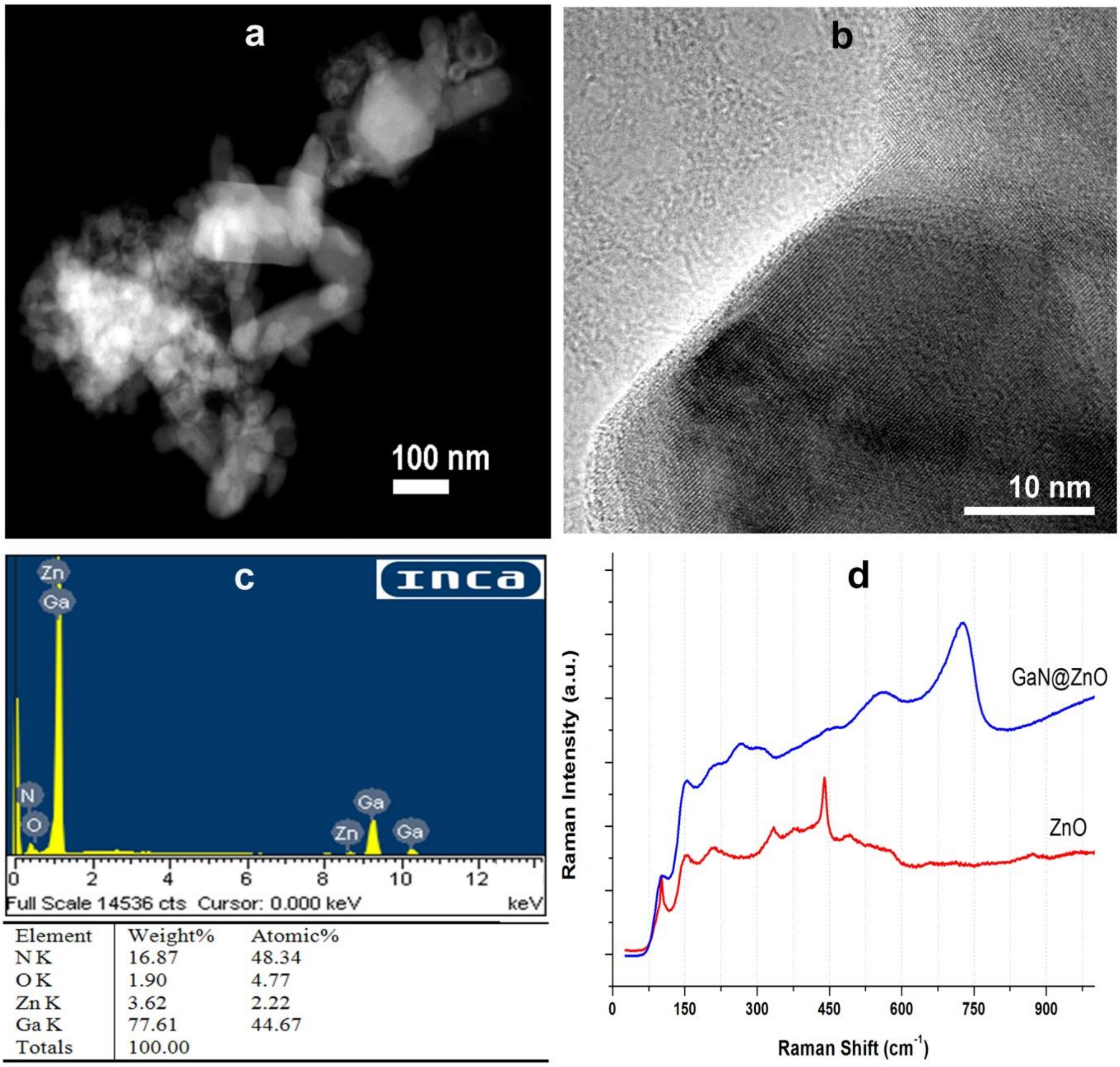

Element	Weight%	Atomic%
N K	16.87	48.34
O K	1.90	4.77
Zn K	3.62	2.22
Ga K	77.61	44.67
Totals	100.00	

Figure S1: TEM images of GaN nanoparticles grown on a sacrificial layer of ZnO nanoparticles (a) and HRTEM of a single nanoparticle (b); EDX analysis is presented in (c) and comparative Raman measurements in (d).

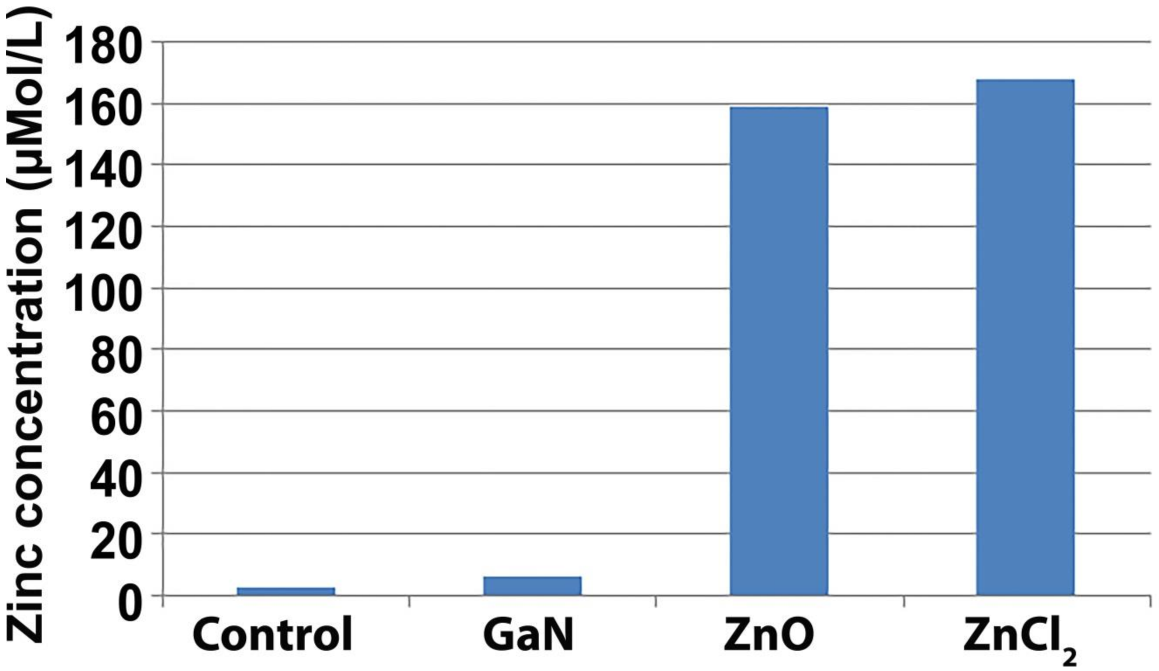

Figure S2: Zinc concentration released in the medium after incubation of EC with different types of nanoparticles at the concentration of 100 µg/mL, positive control is medium without any nanoparticles and negative control are samples with $ZnCl_2$ in the same concentration as ZnO and GaN.

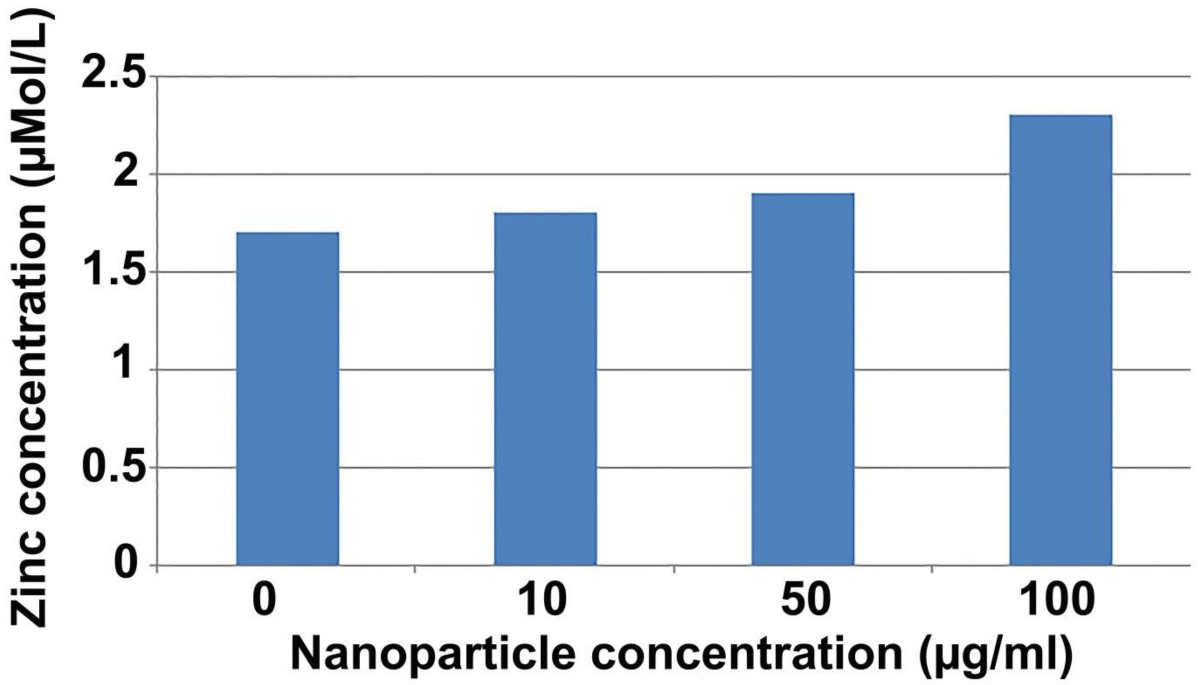

Figure S3: The concentration of zinc released in the culture medium after the incubation of EC with different concentrations of GaN nanoparticles.

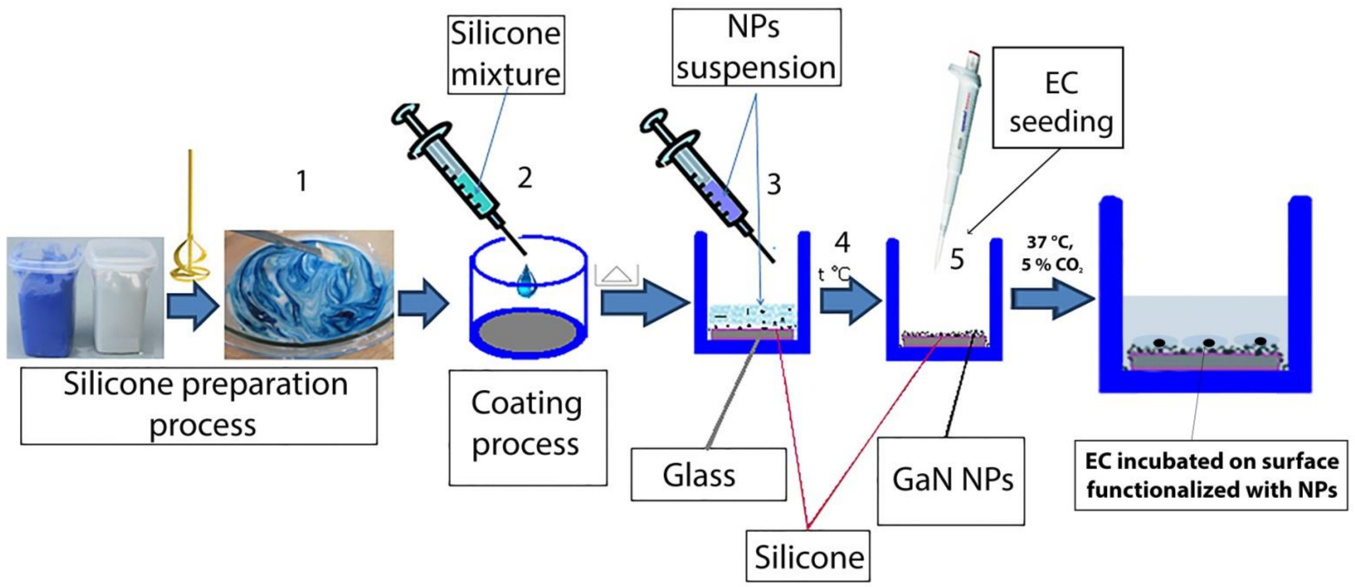

Figure S4: Schematic of the process of surface functionalization with GaN nanoparticles: 1. Mixing the two components of silicone; 2. Silicone spreading on the glass surface by spin coating; 3. Immediately after coating process was done the suspension of nanoparticles in deionized water was added; 4. During 12 h at 60 °C all the water evaporated, after that samples were sterilized at 180°C for 4 h; 5. Mounting coated glasses in culture plates and incubating endothelial cells on them.
